# Scraps

**Published:** 1893-06

**Authors:** 


### SCRAPS

PICKED UP BY THE ASSISTANT-EDITOR.

**A Seasonable Hint.**—*The China Medical Missionary Journal* quotes the following from the *N.-C. Daily News*:

"The little boy doth grapple
With the early summer apple,
And the little boy prevaileth for an hour.
Then the early summer apple
With the little boy doth grapple,
And he fadeth, oh! he fadeth, like a flower."

**Clinical Records** (3).—Patient: "I have had a bottle of port wine sent me. I am not used to it. Do you think I ought to take some?" Doctor: "I don't think port wine is very good for a gouty subject. I don't think you must have any." Patient: "But I never do take any." Doctor: "Very well, then; don't take any." Patient: "But I never have taken any. I am not accustomed to it, aud I don't wish for it." Doctor: "Then we are both agreed, so we won't discuss the point any more." Patient: "But why should you tell me not to take any when you know that I never have taken port wine, and that it does not agree with me?" Doctor changes the subject.

**The Effect of Environment.**—Henrik Dahl, of Aalesund, was a reader and follower of Darwin. Wishing to apply his theory of the limit of adaptability of a species to its environment, he procured a herring from a neighbouring fjord, and carried it home in a tub of sea-water. He renewed the water daily for some time, and gradually reduced the quantity, with so little inconvenience to the herring that he concluded that the fish might, in time, learn to breathe air undiluted with water, like the cat and the man. It turned out as he expected; and the water was finally turned out of the tub never to be replaced. Henrik next removed the fish from its tub, and placed it on the ground, where it flopped about very awkwardly at first, but soon learned to move freely and rapidly. In a little while the herring was able to follow its master without difficulty, and then it became his constant companion about the streets of the city. On a certain unfortunate day Henrik had occasion to cross a dilapidated bridge which spanned an arm of the harbour. The herring, coming gracefully along, heedless of danger, now and again springing at the ephemeræ, for which it had acquired an especial fondness, missed its footing, slipped through a crack into the water beneath, and was drowned.

**The Surgeon.**—Thomas Vicary, "Seriaunt Chirurgion to king Henry the eyght, to king Edward the .vj., to Queene Mary, and to our most gracious Soueraigne Lady Queene Elizabeth, and also cheefe Chirurgion of S. Bartholomewes Hospital," in his well-known book, *A profitable Treatise of the Anatomie of mans body*, notes "foure thinges moste specially that euery Chirurgion ought for to haue: The first, that he be learned; the seconde, that he be expert; the thirde, that he be ingenious; the fourth, that he be wel manered." He deals with these points in detail, and adds that surgeons must "neuer prayse themselues, for that redoundeth more to their shame and discredite, then to their fame and worship; For a cunning and skilful Chirurgion neede neuer vaunt of his dooings, for his works wyll euer get credite ynough. Likewise, that they despise no other Chirurgion without a great cause; for it is meete that one Chirurgion should loue another, as Christe loueth vs all. And in thus dooing, they shall increase both in vertue & cunning, to the honour of God and worldly fame."

The surgical ideal has been somewhat altered since Vicary's time. In "The Ordre of the Hospital of .S. Bartholomewes in Westsmythfielde in London," 1552, it is laid down as an instruction to the surgeons that when they "go to the dressyng of any diseased persone in this house" they "shall geue vnto hym or her, faithfull and good cou*n*saill, willing theim to mynde to sinne no more, and to be thankefull vnto almighty GOD."

With many other things, the "Order" contained the form of "A thankes-geuyng vnto Almyghtie God to be said by the poore that are cured in the hospital, at ye time of their deliuery from thence, vpon their knies in the hall before the Hospiteler, and twoo masters of this house, at the least. And this the Hospiteler shal charge them to learne without the booke, before they be deliuered."

**Medical Philology** (VI).—Following on the notes on "Bledynge boyste," given in March, some further allusions to the practice of bleeding will be of interest.

From a work described as *Mirivalensis in campo florum*, but of which no copy has been found, there came into the *Promptorium* the definition "Bledynge yryn. *Fleosotomium*," or, as it appears in Pynson's printed edition of 1499, *fleobothomium*. In the *Catholicon* the word appears as "Bluderyne" and "Blodeyren," with the Latin equivalents, *fleubotomum*, *lanciola*. Mr. Herrtage's notes are:

'*Phlebotomon.* The instrument to let bloud; a fleume.' Cooper. '*Fleubotomo*; *sanguinem minuere. Fleubotomium: instrumentum cum quo sanguis minuitur.*' Medulla.[1]

In the Invent. of John Stubbes, of York, barber, taken in 1451, we find the following entry: 'De *blode yrens* et launcettes in j case, ij$^{s.}$' *Test. Ebor.* iii. 118.

The *New English Dictionary* gives "lancet" as a definition of "blood iron," a term which, from the entry in Stubbes's Inventory, it would seem not improbable was at one time reserved for the instrument used for scarification with the cupping-glass. In Ambrose Paré's time, as may be seen from an illustration in his book, it was a narrow straight instrument, with one end bent to form a small handle, and a protruding cutting edge at the other. While the form of the cutting instrument used in cupping has greatly changed since Paré's day, the lancet, of which he also gives a drawing, was practically the same shape as at present.

The speciality in which these instruments were used was subdivided into the drawing of blood by venesection and the practice of cupping. In an English Vocabulary of the fifteenth century, printed by Wright (*Anglo-Saxon and Old English Vocabularies*, 2nd ed., 1884, I. 652), "*Hic fleobotomator*, $A^e$ blodelater" and "*Hic scarificator*, $A^e$ carsare" are given under "Nomina Artificiorum." Wright had not met with the word "carsare" before, and he offers no explanation of it. It is not given in the *New English Dictionary*. The word may by some transposition of letters, or by miswriting, be connected with *scaro*, which in the March *Journal* I quoted from the *Promptorium*, where, with *ventoso*, it is given as one of the Latin equivalents of "boyston."

*Ventose* became the technical name in English for the cupping-glass, and is used in the 1632 translation of Paré. Chaucer, in describing the nature of Arcite's fatal injuries, says (*Knightes Tale*, ll. 1887–90):

"The clothered blood, for any lechecraft,
Corrupteth, and is in his bouk y-laft,
That nother veyne-blood, ne ventusinge,
Ne drinke of herbes may ben his helpinge."

In modern French, *Ventouse* is the word for cupping-glass.

In earlier times the vessel used was made of brass or horn. Celsus (II. xi) says: "Cucurbitularum vero duo genera sunt: aëneum, et corneum. Aënea, altera parte patet; altera, clausa est: cornea, altera parte æque patens, altera foramen habet exigium. In aëneam linamentum ardens conjicitur, ac sic os ejus corpori aptatur, imprimiturque, donec inhæreat. Cornea per se corpori imponitur; deinde, ubi ea parte, qua exiguum foramen est, ore spiritus adductus est, superque cera cavum id clausum est, æque inhærescit." Paré refers to the horn, and describes in detail the same mode of using it.

1 This is the *Medulla Grammatice*, the earliest known Latin-English Dictionary, and supposed to have been compiled by the writer of the *Promptorium*. There are several versions of it among the Harleian MSS. in the British Museum. There are also, besides those in some private libraries, copies in the Cathedral libraries of Canterbury and Lincoln, and in the library of King Edward's Grammar School at Shrewsbury. All of them were written in the latter half of the fifteenth century.

